# Aging Mechanism and Rejuvenating Possibility of SBS Copolymers in Asphalt Binders

**DOI:** 10.3390/polym12010092

**Published:** 2020-01-04

**Authors:** Fusong Wang, Lei Zhang, Xiaoshan Zhang, Hechuan Li, Shaopeng Wu

**Affiliations:** 1 State Key Laboratory of Silicate Materials for Architectures, Wuhan University of Technology, Wuhan 430070, China; wangfs@whut.edu.cn (F.W.); zxs971206@163.com (X.Z.); wusp@whut.edu.cn (S.W.); 2 Department of Civil and Environmental Engineering, Norwegian University of Science and Technology, 7491 Trondheim, Norway; lei.zhang@ntnu.no

**Keywords:** SBS copolymers, penetrative rejuvenator, asphalt binder, ultraviolet aging, rheological property

## Abstract

The styrene–butadiene–styrene (SBS)-modified asphalt pavement has been in growing demand in the road construction field owing to its workable mechanical property and temperature durability. This paper prepared a penetrative rejuvenator (PR) with waste cooking oil (WCO) and emulsified asphalt, then applied PR on SBS copolymers to investigate its aging and rejuvenating effects in an asphalt binder. After a thin film oven test (TFOT) and ultraviolet (UV) aging of SBS copolymers, Fourier transform infrared (FTIR) spectra were used to analyse the aged copolymers’ chemical structure. Moreover, both aged and rejuvenated SBS copolymers were added into a fresh asphalt binder to get two kinds of modified asphalt binders, namely, MAAC (modified by aged copolymer) and MARC (modified by rejuvenated copolymer). Aiming to analyse the monomer effect of SBS copolymers in the asphalt binder, the rheological characteristic with dynamic shear rheometer (DSR), chemical structure with FTIR and physical properties with penetration, soft point and ductility tests were investigated using MAAC and MAAC samples. The results showed that rejuvenated SBS copolymer could improve MAAC’s viscoelasticity, but from FTIR spectral analysis, PR resulted in no chemical changes to SBS copolymers. A tough coat which made MAAC of higher stiffness was observed on the copolymer surface after thermal treatment. UV caused evidently negative effects on SBS copolymer because of accelerating oxidation by ozone, which brought about high possibility of cracks during servicing periods of asphalt pavement. In addition, MAAC was inferior in both rheological and physical properties, which reflected the significance and necessity in consideration of alleviating SBS copolymer aging in field.

## 1. Introduction

With the rapid development of pavement construction, SBS-modified asphalt is widely used in the upper layer or covering layer of pavements around the world [1,2,3]. Since a copolymer phase is finely dispersed in the asphalt binders, SBS-modified asphalt pavement can relieve the stress cracking in low-temperature and improve the rutting resistance in thermal conditions [4,5,6,7]. However, it is acknowledged that several inevitable factors could deteriorate the rheological property of SBS-modified asphalt binders [8,9], such as the heating age during mixing and paving, heavy traffic load and extreme service environments, leading to some pavement distresses appearing thereby. Meanwhile, SBS copolymers could also tend to be aged by exposing to outside heat, oxide [10] and ultraviolet light due to the sensitivity of the double bonds of polybutadiene segment [11], which may result in discoloration and surface embrittlement, subsequently impacting its macroscopic physical properties [12].

As the situation that aged SBS copolymers would trigger considerable adverse effects in whole SBS-modified asphalt system [7], numerous scholars have conducted associated investigations in order to analyse the monomer effect of aged SBS copolymers. Gao [13] analyzed the thermo-oxidative aging process of SBS-modified asphalt. The result of FTIR and gel permeation chromatography (GPC) tests showed that SBS copolymers were severely degraded after aging treatment. Moreover, its long-term aging could cause an invalid application for SBS-modified asphalt. Larsen [2] blended two base asphalts and two different SBS copolymers under different experimental conditions, and found that the high shearing rate and high mixing temperature caused SBS copolymers degradation. This reference concluded that the degradation fragments would decrease the viscosity of SBS-modified asphalt. Cortizo [14] investigated three types of SBS copolymers with a rolling thin film oven test (RTFOT) and pressure aging vessel (PAV). The results indicated that the structural characteristics of copolymer and aging conditions would influence physical and rheological properties of SBS-modified asphalt. Maria [15] aged the SBS copolymers in inert and oxidative atmospheres, respectively, and found that oxidation resulted in the increasing of carbonyl, hydroxide and sulphoxide groups. Wang [16] analysed the aging mechanism of SBS-modified asphalt with chemical reaction kinetics; the result showed that the network structure of SBS copolymer was lost gradually as short-term aging degrades process, and the activation energy became lower. Similarly, Xu [17] also studied its thermal oxidation mechanism and divided the self-catalysed process into four steps to consume the active centres in SBS copolymers. It can be concluded that the thermo-oxidative aging (TFOT and RTFOT) process of SBS copolymer was usually studied in this field, but the ultraviolet aging process was seldom covered at present. Since the SBS copolymer mainly exists in the physical expansion of asphalt, it is necessary to analyse the aging properties of SBS copolymer in consideration of the improvement of overall modified asphalt.

Besides the aged properties of SBS copolymer and SBS-modified asphalt, their rejuvenated measures and mechanism also raise some concerns. Cong [18] blended rejuvenator, fresh and reclaimed SBS asphalt binder to investigate the regeneration effects. Zhang [19] proposed that 9 wt % dosage of rejuvenator could obtain a similar physical property with original SBS-modified asphalt. Xu [20] prepared a reactive rejuvenator containing epoxy functional groups which was able to react with carboxylic groups to bond those oxygenated chain fragments formed in the degradation of SBS. Guo [21] used FTIR to analyse the inner structure of SBS-modified asphalt systems, and results demonstrated that modification effects of SBS copolymers were gradually lost after aging. Xu [22] utilized FTIR and X-ray photoelectron spectroscopy to spied the changes on rejuvenated SBS copolymers after being treated with self-designed rejuvenator. Nevertheless, with the rising reports demonstrating that preventive maintenance was the effective and environment friendly alternative to improve servicing properties and prolong the servicing life of asphalt pavement [23,24], the majority of scholars conducted research on preventive maintenance for SBS asphalt pavements [25,26]. Fog seal, one of the preventive maintenance approaches has attracted increasing attention in scientific research and engineering applications in the asphalt pavement field [27,28,29]. It means spraying the rejuvenators on the pavements to alleviate or even heal pavement slight diseases efficiently in its early occurrence [30]. Accordingly, investigating the rejuvenation of SBS asphalt pavement should not only tackle the challenge in performance degradation of SBS-modified asphalt binder, but enhancing the rejuvenating effects on SBS copolymers also need sufficient explorations.

This study prepared a kind of penetrative rejuvenator (PR) with waste cooking oil and emulsified asphalt. Besides that, our experiments aged the SBS copolymers with thermal oxygen and ultraviolet conditions to get different samples, and analysing appearance changes and microscopic composition after aging and rejuvenating treatments. In relation to the SBS-modified asphalt, the aged SBS copolymers were sheared with base asphalt to analyse the individual effects of an aged SBS copolymer, their physical and rheological properties, as well as the samples’ chemical structure. The study will probably be a significant reference in further applications of SBS-modified asphalt.

## 2. Materials and Experiments

### 2.1. Materials

**Figure 1:**
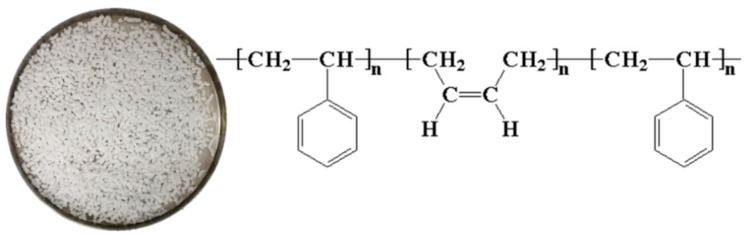
Appearance of original SBS copolymer.

SBS copolymers (YH 792), supplied by Sinopec (Sinopec Baling Petrochemical Co., Ltd, Hunan, China), was used as asphalt modifiers. Figure 1 depicts the appearance and organic chemical structure of original SBS copolymers [9], and its basic properties is listed in Table 1. AH 90 asphalt binder was generally used as raw materials for modified asphalt owing to its sufficient content of light components [6,31], and this study also chose 90-grade heavy traffic asphalt (Hubei Guochuang Hi-tech Material Co., Ltd., Wuhan, China) to mix with SBS copolymers for further experiments, and Table 2 listed its fundamental properties.

### 2.2. Experiments

**Figure 2:**
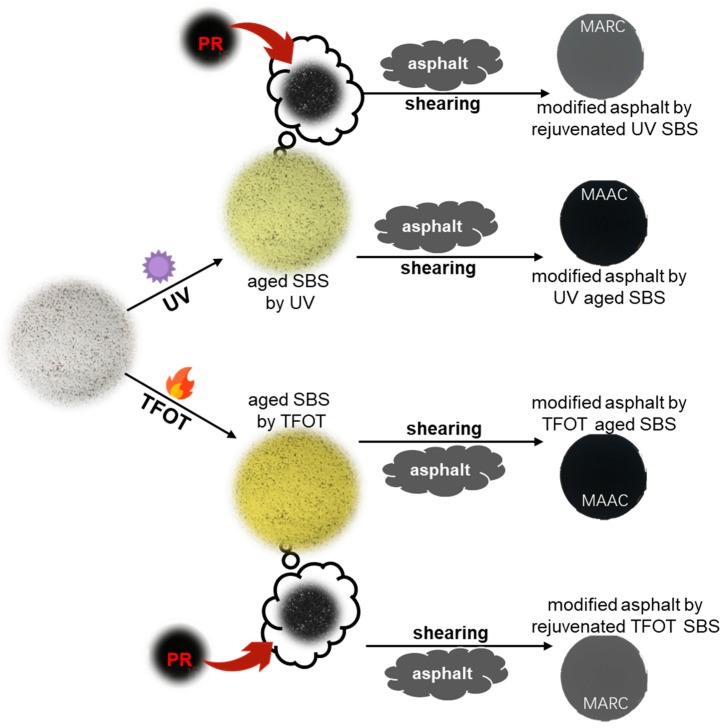
Research flowchart of experiments.

The study analyzed the adverse effects of UV and thermal factors on chemical structures of the SBS copolymer. Then, PR (preparing steps are depicted in Figure 2) was proposed to evaluate the copolymers’ rejuvenating influences. After that, different aged SBS copolymers were sheared with fresh asphalt for MAAC samples. Then, the physical and rheological properties of modified samples were tested. Moreover, the study rejuvenated MAAC samples with PR to obtained MARC samples. Via the comparison with MAAC and MARC in rheological properties and chemical structure, the study aimed to evaluate the monomer effects of aged and rejuvenated copolymers in whole modified asphalt system. Figure 2 explains the experimental flowchart.

#### 2.2.1. Preparation of Penetrative Rejuvenator

**Figure 3:**
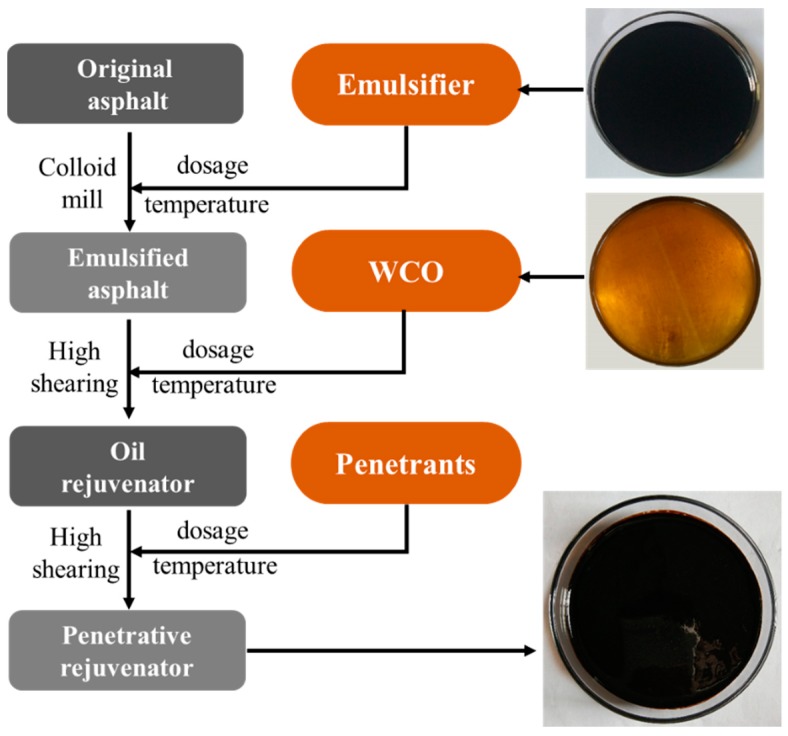
Process schematic diagram and the row materials.

According to the fog seal in asphalt pavement maintenance, a penetrative rejuvenator (PR) was self-designed and prepared in the laboratory with waste cooking oil (WCO, Guisheng Oil Materials Co., Ltd., Dongguan, China) and emulsified asphalt [32]. Penetrants which were produced by fatty alcohol polyoxymethylene ether and ethylan 1005 (nonionic surfactant based on a synthetic primary alcohol) were added to improve the permeability, while being sprayed onto aged asphalt pavement surface [33]. The schematic diagram of the PR preparation process and the used raw materials are shown in Figure 3. Firstly, emulsified asphalt was produced by colloidal mill, then, a mass ratio of 60% emulsified asphalt and 40% WCO were added into a high shearing machine for oil rejuvenator (5000 rpm, 120 min). Finally, a mass ratio of 0.8% penetrants was introduced into the emulsion at 85 °C to produce PR (5000 rpm, 30 min). Table 3 shows the fundamental properties of PR. The analysis of chemical SARA compositions [34] demonstrated that the high content of saturates and aromatics could have good effects on adjusting compounds of aged asphalt [35].

#### 2.2.2. SBS Copolymers

SBS copolymer is known for its high elasticity and good fatigue resistance when used to modify asphalt in the construction field [14]. Accordingly, SBS copolymers in this part were handled with two kinds of aging treatments, respectively. The thermal aging was considered by TFOT (standard ASTM D1754 [36]), and ultraviolet aging by UV (standard ASTM G154-16 [37]). In order to analyze upon appearance changes and chemical composition, the related aging time and temperature in aging treatments were controlled as listed in Table 4. The referenced literature [22] recommended that 20 wt % of rejuvenator dosage for SBS copolymers could achieve an acceptable rejuvenating effect. In order to fully rejuvenate aged SBS, a mass ratio of 20% PR was sprayed on the surface of 80% aged copolymers, then it was placed into an experimental dryer at room temperature (25 °C) for 24 h to get rejuvenated SBS copolymers. Table 4 also shows rejuvenated sample labels.

**Figure 4:**
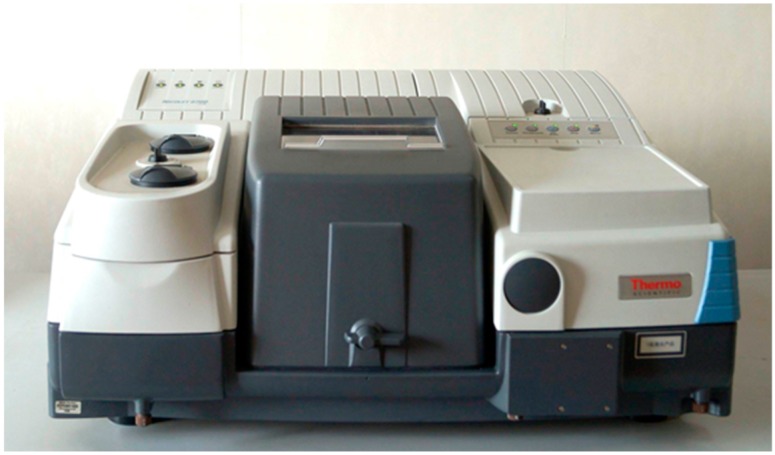
Photograph of the FTIR used in this work.

**Figure 5:**
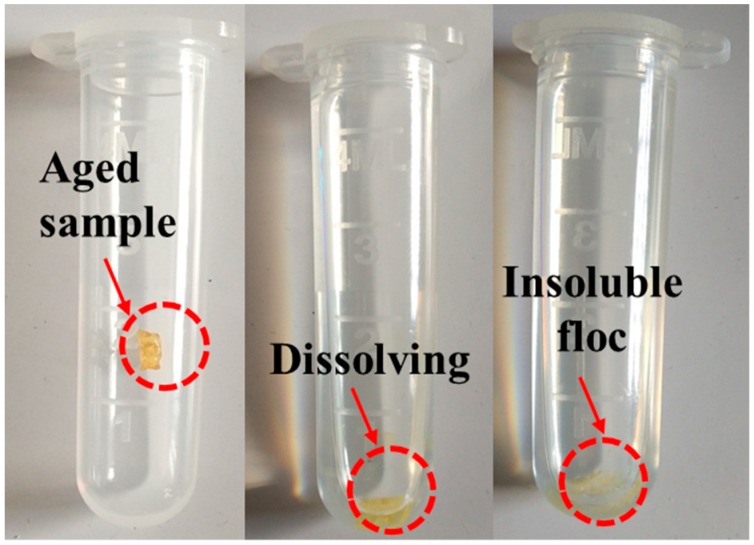
Insoluble yellow flocs.

FTIR spectra were used to characterize the chemical structure changes after samples went through TFOT and UV-accelerated aging. Figure 4 shows the FTIR instrument, whose specification is Nicolet 6700 (Thermo Electron Scientific Instruments, Madison, WI, US). The wavenumber range is 400–4000 cm^−1^ and 64 scans were conducted to improve the signal-to-noise ratio per spectra. In addition, some yellow flocs could not be dissolved during dissolving aged SBS copolymers with carbon disulfide, as shown in Figure 5, while the original SBS could be dissolved easily in the same condition. The phenomenon indicated that some new and different substances in copolymers were formed after the aging reaction. Therefore, the FTIR samples were produced with the pressing plates method.

#### 2.2.3. SBS-Modified Asphalt

Five aged SBS copolymer-modified asphalt samples were obtained, respectively, from the five types of aged SBS copolymers that are described in Table 4, which were prepared from shearing aged copolymers and original asphalt with a high shearing machine (5000 rpm, 180 °C, 90 min, ELE Mechanical & Electrical Equipment CO., LTD., Shanghai, China). Similarly, five correspondingly rejuvenated samples were prepared at the same shearing temperature and rate by rejuvenated SBS and fresh asphalt. On the basis of this, original asphalt and unaged SBS-modified asphalt were investigated as a control group. The samples were labeled as Table 5.

In consideration of the physical properties of asphalt samples, penetration (25 °C), ductility (5 °C) and softening point were tested, which presented significant references and empirical values related to comprehensive road performance in actual service time. Then, the rheological properties of modified asphalt samples were studied through temperature sweep in dynamic shear rheometer (DSR, Anton Paar, Shanghai, China). According to the complex shear modulus (G*) and phase angle (δ), the rutting factor (G*/sinδ) of different samples was calculated and analyzed. Moreover, the fatigue property of different samples was measured under 3% strain level controlled condition with DSR at a frequency of 10Hz and temperature of 20 °C, which played a significant reference in illustrating asphalt pavement durability in servicing life [38]. Finally, FTIR was used to analyze the chemical structure of MAAC and MARC samples. After being dissolved in carbon disulfide, MAAC and MARC samples tested at a same condition with former SBS copolymers. The spectra data were analyzed by software (*OMNIC*, Thermo Fisher Scientific, Branchburg, NJ, US) [39], besides, the structural indices of chemical groups were referred to make quantitative analysis in aged and rejuvenated effects [40].

## 3. Results and Discussions

### 3.1. Characteristics of Aged SBS Copolymers

**Figure 6:**
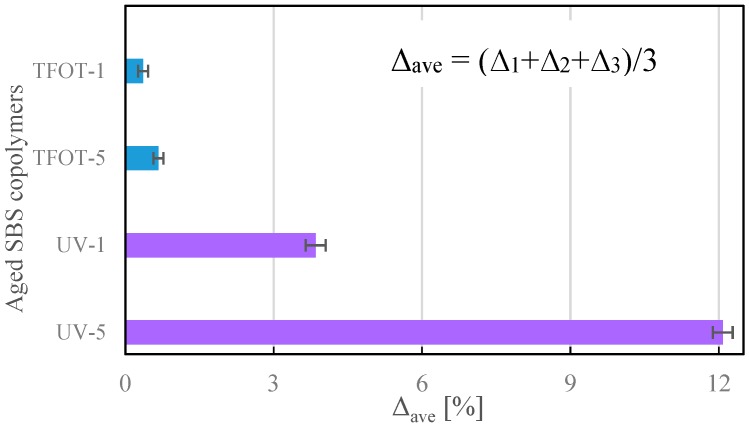
Average value in quality changes.

The mass changes of copolymers were recorded during aging, represented with Δ, whose calculating principle is shown in Equation (1). Their average value, Δ_ave_, was generated from three parallel groups for every label. Analyzing the results depicted in Figure 6, it was distinct to find that all copolymers’ masses increased gradually during UV and TFOT treatment. Moreover, the longer aging time generated, the greater mass gained. It was demonstrated that the copolymers had a reaction with the oxygen from atmosphere. More specifically, the unstable groups were oxidized to oxygen-containing groups, such as hydroxyl, carbonyl and ether bonds. Hence, as for SBS copolymers at the same aging condition, the amount of increased mass could reflect the degree of oxidation, and that of the aged situation in general.

Meanwhile, the Δ_ave_ of UV aged copolymers was much larger than that of TFOT, which meant that the copolymers were more thoroughly oxidized in UV conditions. This phenomenon could be explained with Equation (2). Irradiated by ultraviolet light in the aging box, parts of oxygen were transformed into ozone [41], which behaved stronger in terms of oxidizability. Consequently, SBS copolymers were oxidized by both ozone and oxygen in the ultraviolet aging box, the larger then was obtained at a UV aging condition.

(1) $$\Delta =\frac{m2-m1}{m1}\times 100\%$$

where Δ is the percentage change in mass of tested sample, %; m1 is the mass of sample before being aged, g; m2 is the mass of sample after aged, g.

(2) $$O_{2}\overset{UV}{\to }O_{3}$$

**Figure 7:**
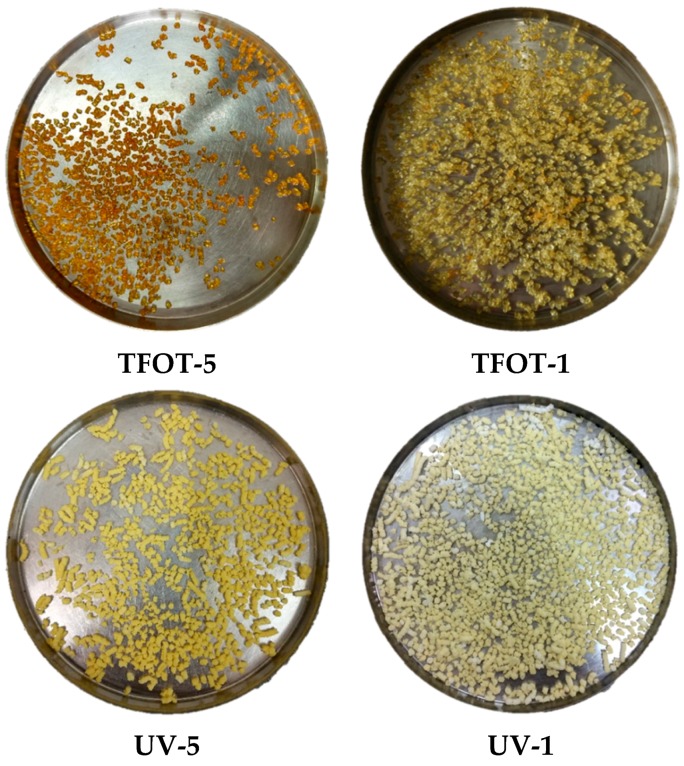
Appearance of aged SBS copolymers.

Figure 7 shows the appearance of aged SBS copolymers. Compared to the original SBS copolymer in Figure 1, it was obvious that all aged copolymers became yellow, and a darker color appeared with a longer aging time. In addition, the morphological characters of SBS copolymers had changed with the thermal condition, which appeared as a melting phenomenon around the copolymers and glued to the sample plate. As to the UV-aged samples formed, the morphological characters had not changed regardless of the color. It was postulated that the high temperature caused copolymer melting gradually, then, copolymers surface became dense and stiff.

**Figure 8:**
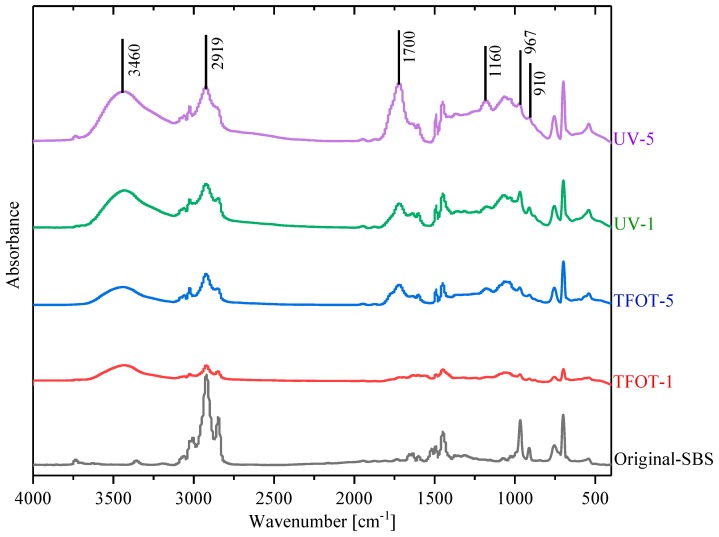
The spectra of aged copolymers.

Including PR and rejuvenated copolymers, there were ten samples tested with FTIR in this study. Relevant partial peaks, assignments and functional groups in the spectrum are summarized in Table 6 [10]. Figure 8 illustrates the spectra of aged copolymers and unaged SBS. It indicates that the absorption peak of a double bond (967 cm^−1^) had an obviously decreasing trend after being aged. Nevertheless, the new absorption peaks of hydroxyl (3520 cm^−1^), carbonyl (1725 cm^−1^) and ether bonds (1160 cm^−1^) appeared in aged samples. That means parts of the double bonds degraded during aging, and oxidized to oxygen-containing groups for hydroxyl, carbonyl and ether bonds. Accounting for the peak of hydroxyl (3520 cm^−1^), it was obvious that UV-5 had the strongest peak, and UV-1 geo the next, corresponding to the conclusion of Figure 6 that ultraviolet-worsened copolymers statement more significantly, although UV and high temperature (163 °C) could both lead to a serious aging of SBS copolymer.

**Figure 9:**
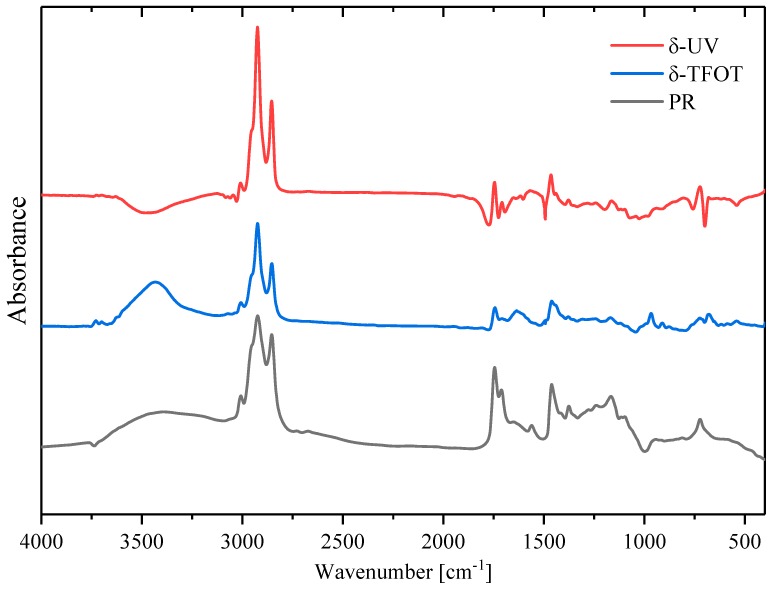
Comparison of PR spectra and subtracted spectra (δ-UV and δ-TFOT).

The software *OMINIC* was utilized to calculate the difference spectra between two different samples in this experiment. The difference spectra (δ-UV) by UV-5-R subtracting UV-5 and the difference spectra (δ-TFOT) by TFOT-5-R subtracting TFOT-5 were obtained and compared with the PR spectra roughly, as shown in Figure 9. It can be observed that absorbance peak intensity was different in the same wavenumber range. It was explained that different PR dosages were applied to aged copolymers, which contributed to the different absorbance peak intensity. Similar spectra trends in Figure 9 indicated that PR did not have a chemical reaction with SBS copolymers. 

SBS copolymers could absorb the oil compounds and swell in asphalt dispersedly [42]. PR is abundant in waste cooking oil and emulsified asphalt, which cannot make a chemical reaction. This reflected that oil generated no reaction with SBS copolymers. On the other hand, FTIR spectra results indicated that the rejuvenator could only have a positive influence on asphalt rather than a single SBS copolymer.

### 3.2. Characteristics of SBS-Modified Asphalt

#### 3.2.1. Physical Properties Analysis

**Figure 10:**
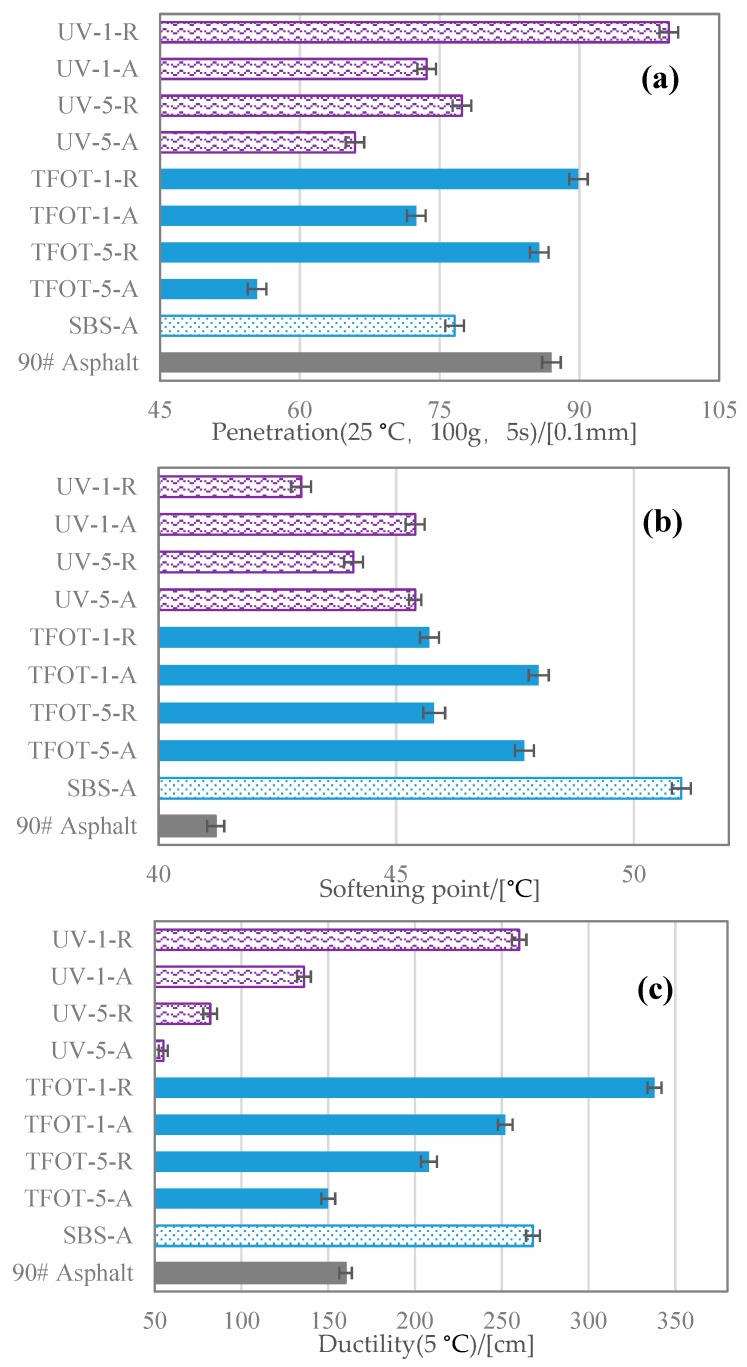
Physical properties of MAAC and MARC samples: (**a**) penetration; (**b**) softening point; (**c**) ductility.

Figure 10 illustrates the physical properties of ten samples, involved in their penetration, softening point and ductility. Referring to the results of the penetration test (Figure 10a), it can be noted that the addition of SBS copolymer led to a lower penetration value, and the aged SBS copolymers with thermal and ultraviolet treating also caused a lower penetration value compared with original SBS. The trend reflected that aged copolymers brought worse viscoelasticity to modified asphalt. Nevertheless, rejuvenated copolymers by PR could recover the declined penetration value of MAAC samples apparently. As for softening point test (Figure 10b), the addition of SBS copolymers gave rise to a substantial increase for original asphalt in softening temperature. The difference between various MAAC samples was unnoticed; moreover, rejuvenation by PR also had a slight influence in softening points of MAAC samples. That meant SBS copolymers could promote asphalt thermal performance, while aged SBS copolymers and treating PR would make weaker impacts on its high temperature property. According to the ductility test results (Figure 10c), addition of SBS copolymers had promoted the tensile strength in low temperature. While aged SBS copolymers had decreased the ductility with an evident scale in contrast, especially for the ultraviolet aging which led to an obvious decline compared to the base asphalt value. Yet, PR could still refresh the sample ductility with a large extend.

Consequently, aged SBS copolymers would result in quite a few demerits for the physical properties of modified asphalt. For instance, lacking sufficient ductility might raise the possibility in generating cracks as applied in lower temperature particularly. Based on the previous result that UV contributed to considerably inferior effects on SBS copolymers, correspondingly, UV MAAC also caused more profound deterioration in physical properties. Additionally, PR can enhance MAAC samples’ rheological characteristics generally.

#### 3.2.2. Rheological Properties Analysis

**Figure 11:**
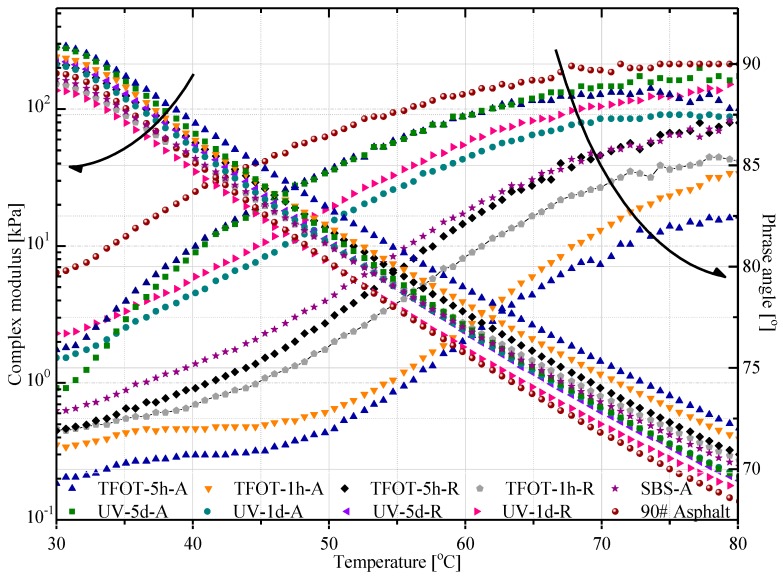
Changes of complex modulus and phase angle.

Mixing asphalt with aged and rejuvenated copolymers, nine types modified asphalt and another base asphalt sample were tested by DSR with 10 rad/s of angular frequency and a temperature range of 30–80 °C. Figure 11 shows each sample’s complex modules and phase angles. Evidently, TFOT-5-A had the maximum complex modules and minimum phase angle during the measuring temperature, which meant it had a bigger stiffness in responding the shear. Then, TFOT-1-A had the second smallest phase angle, yet, UV-5-A had a bigger module than that of TFOT-1-A at the temperature range of 30–40 °C. It could be explained that the surface of SBS copolymer was melted slightly as it was experiencing thermal conditions, which resulted in a hard layer coating, then higher stiffness leading to the rising of a complex module in modified asphalt.

Besides, the MACR samples displayed a smaller complex modulus and bigger phrase angle in general. Specifically, the PR contain sufficient oil components which can adjust and reconcile the composition of modified asphalt in certain extent. Consequently, it provided the improvement in modified asphalt rheological characteristic, but it may also increase the rutting possibility in field application. Regarding to the servicing performances of SBS-modified asphalt pavement, PR could supply light componence (oil content) and making up its viscosity loss, then enhancing the anti-cracks property in low temperature condition. 

**Figure 12:**
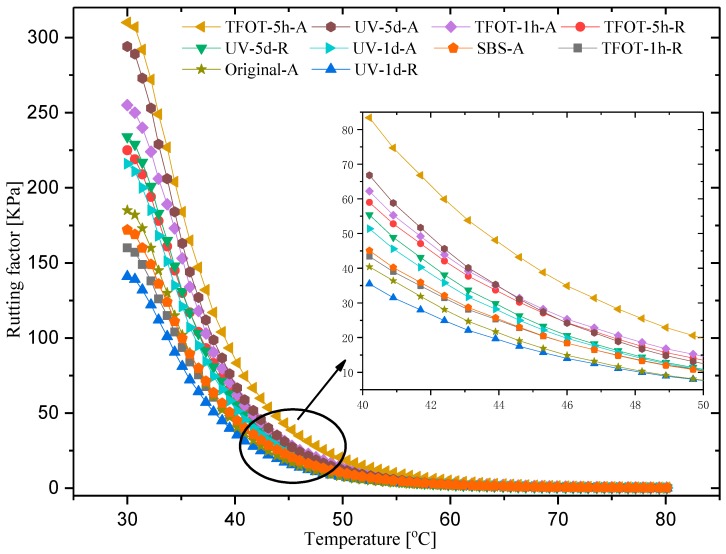
Rutting factor of different samples.

Considering phase angle and complex module synthetically, a rutting factor can be obtained to evaluate samples’ rutting resistance [43]. The relevant curves shown in Figure 12 demonstrate that TFOT-5-A had a maximal rutting factor, meanwhile, UV-5-A and TFOT-1-A followed up with a similar pace at temperatures over 43 °C. Partial enlargement detail in Figure 12 provided distinct vision within 40–50 °C. Comparably, SBS-A got a greater rutting factor value than base asphalt, while the rejuvenated sample obtained lower rutting resistances. This indicated that SBS copolymer could enhance the rutting resistance remarkably, while PR were able to promote the rheological characteristic of MAAC samples, with a rising rutted potential correspondingly. UV-5-A was severely aged according to former FTIR spectra results, but a lower rutting factor appeared in Figure 12. Therefore, both aged approaches and conditions of SBS copolymer might be associated with the rutting factor directly, instead of a single aged degree.

**Figure 13:**
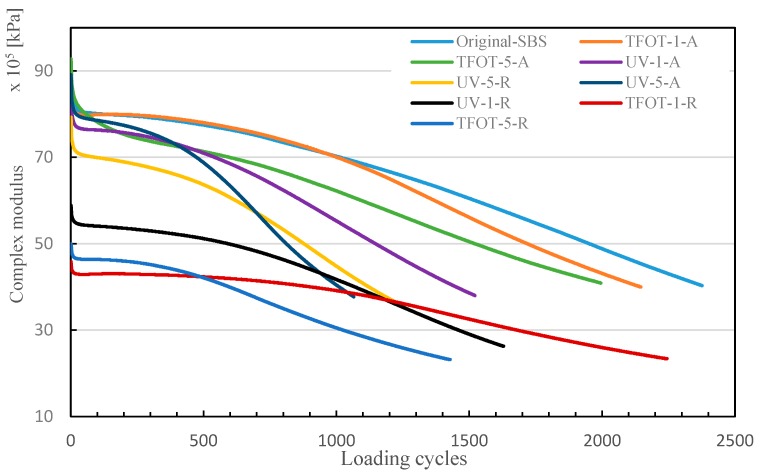
Fatigue life of different samples.

Additionally, the study measured the samples’ fatigue life with DSR at a frequency of 10 Hz and temperature of 20 °C, and the results are shown in Figure 13. The number of cycles with 50% reduction of initial complex modulus was used to evaluate the samples’ fatigue life. It is noticed that all rejuvenated samples had a lower complex modulus, and Original-SBS had the longest loading cycles. That meant the aged SBS copolymer could cause fatigue life shortening in modified asphalt. Although rejuvenator decreased modified asphalt complex modulus, its loading cycles were increased evidently. Besides, the fact that UV aging samples showed shorter loading cycles demonstrated that UV could bring about worse aged condition for SBS copolymers than thermal conditions. Hence, MAAC by ultraviolet would result in the fatigue life of asphalt pavement decreasing rapidly.

#### 3.2.3. Chemical Structure Integration

**Figure 14:**
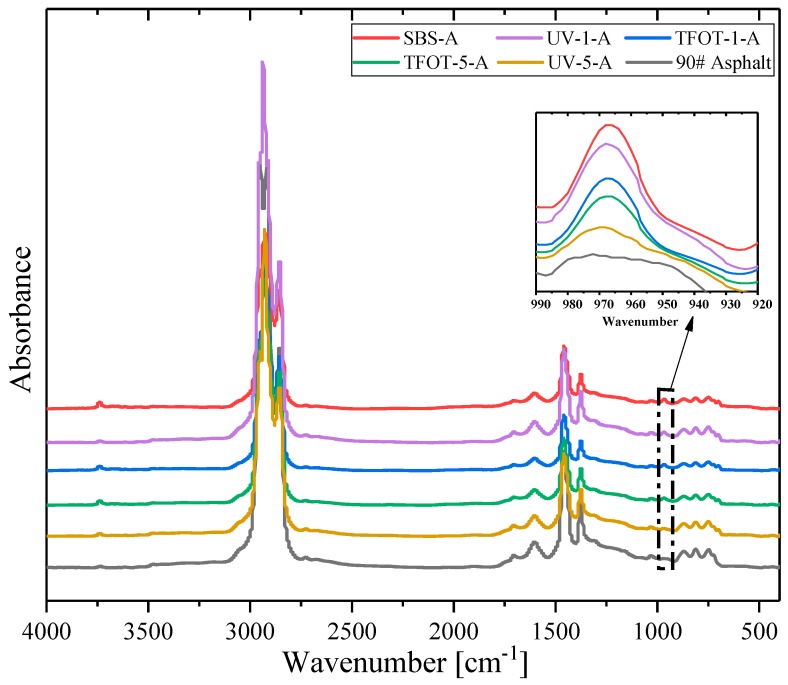
FTIR spectra of aged samples.

It is recognized that the absorption peak of a double bond (967 cm^−1^) is a fingerprint of SBS copolymers, while carbonyl (1700 cm^−1^) and sulfoxide (1300 cm^−1^) groups are referenced as the reaction between active molecules in asphalt and oxygen in air during the aging process [44]. Therefore, the experiment studied the inner molecular transformation of SBS copolymers and its modified asphalt by calculating characteristic peak intensity, respectively. As shown in Figure 14, the FTIR spectra of MAAC samples have similar flow trends. The intensity difference of the absorbance peak in a double bond was amplified in Figure 14, which demonstrated that UV-5-A got a deeper aged degree than TFOT-5-A, but TFOT-1-A showed a deeper aged degree than UV-1-A. That meant a higher aged statement in SBS-modified asphalt could be achieved when exposed to UV for a long term. High temperature might also lead to its severe aged condition during a short time (5 h). In addition, the thermal condition (163 °C) was unreachable in practical servicing time, however, constantly exposing asphalt pavements to UV would be accessible in the field. Therefore, UV may be a principally environmental factor which needs to be taken into consideration for SBS-modified asphalt construction in actual life.

In the basis of FTIR spectra, the study investigated modified binders’ relative intensity and structural indices at three characteristic absorption peaks: double bond, carbonyl and sulfoxide group, listed in Table 7. The Equations (3)–(5) expressed their calculation principles, respectively. Evidently, SBS-A had a high double bond content, and its amount declined gradually as aging treatment proceeded, but MARC samples has a higher intensity for double bonds. Because PR contained parts of double bond components in the oil ingredient, the structural index of sulfoxide group was higher in aged samples with the vulcanization of the addition of sulfur in SBS copolymer. Another noticeable trend was that ultraviolet-aged samples obtained a higher carbonyl and sulfoxide indices than TFOT samples, which was explained by the fact that ultraviolet accelerates the oxidation reactions in the existence of ozone, more carbonyl and sulfoxide groups were then generated. Hence, UV could result in more considerable adverse effects in the service performance of SBS-modified asphalt pavements than other factors. Moreover, MARC samples displayed lower sulfoxide content and higher carbonyl content, which illustrated that PR had contained several carbonyl contents and fewer or no sulfoxide groups. So structural indices of rejuvenated samples cannot reflect their chemical structure accurately.

(3) $$I_{C=O}=\frac{\mathrm{Area}\;\mathrm{of}\;\mathrm{the}\;\mathrm{carbonyl}\;\mathrm{band}\;\mathrm{centered}\;\mathrm{around}\;1700{\;\mathrm{cm}}^{-1}}{\mathrm{Area}\;\mathrm{of}\;\mathrm{the}\;\mathrm{spectral}\;\mathrm{bands}\;\mathrm{between}\;2000{\;\mathrm{cm}}^{-1\;}\mathrm{and}\;600{\;\mathrm{cm}}^{-1}}$$

(4) $$I_{S=O}=\frac{\mathrm{Area}\;\mathrm{of}\;\mathrm{the}\;\mathrm{carbonyl}\;\mathrm{band}\;\mathrm{centered}\;\mathrm{around}\;1030{\;\mathrm{cm}}^{-1}}{\mathrm{Area}\;\mathrm{of}\;\mathrm{the}\;\mathrm{spectral}\;\mathrm{bands}\;\mathrm{between}\;2000{\;\mathrm{cm}}^{-1\;}\mathrm{and}\;600{\;\mathrm{cm}}^{-1}}$$

(5) $$I_{CH=CH}=\frac{\mathrm{Area}\;\mathrm{of}\;\mathrm{the}\;\mathrm{carbonyl}\;\mathrm{band}\;\mathrm{centered}\;\mathrm{around}\;967{\;\mathrm{cm}}^{-1}}{\mathrm{Area}\;\mathrm{of}\;\mathrm{the}\;\mathrm{spectral}\;\mathrm{bands}\;\mathrm{between}\;2000{\;\mathrm{cm}}^{-1\;}\mathrm{and}\;600{\;\mathrm{cm}}^{-1}}$$

## 4. Conclusions

The appearance characteristics and chemical structure changes of SBS copolymers were studied before and after UV and TFOT treatments. With the addition of aged and rejuvenated SBS copolymers, respectively, their associated monomer effects on asphalt binder’s physical and rheological properties were also analyzed. Afterward, FTIR was utilized to obtain the chemical structural information of MAAC and MARC samples via spectra and structural indices analyses. Based on the discussed results, the following items can be concluded:

(1) SBS copolymer could be easily aged in thermal and UV conditions from oxidation reactions, accompanied with their quality increasing and yellowing as they age. Besides, the ozone generated from the ultraviolet circumstance played a critical effect in the aging process.
(2) Partial double bonds in SBS copolymers were degraded during aging, and oxidized to oxygen-containing groups, such as hydroxyl, carbonyl and ether bonds. Nevertheless, rarely evident chemical reactions take place between PR and SBS copolymer.
(3) Aged SBS copolymers would deteriorate the physical properties of modified asphalt with a higher possibility in generating cracks at lower temperature, as well as shorter fatigue life by considering the changes of rheological properties.
(4) UV would be a major adverse factor in the aging SBS copolymer and brings about servicing time decrease in the field. Although PR contains sufficient oil components which can alleviate the negative influences in aged SBS asphalt with adjusting the composition of asphalt binder, PR has little rejuvenating effects in SBS copolymers directly.

## Figures and Tables

**Table 1:** Basic properties of SBS copolymer.

Sample	S/B Ratio	Volatiles [%]	Total Ash [%]	Tensile Strength [MPa]	Density [g/cm^3^]
SBS copolymer	40/60	<0.7	<0.2	>24.0	1.32

**Table 2:** Fundamental properties of 90-grade heavy traffic asphalt.

Items		Results
Physical properties	Penetration (25 °C) [0.1 mm]	80.5
	Ductility (10 °C) [cm]	>100
	Softening point [°C]	41.3
	Viscosity (135 °C) [cP]	533
Chemical compositions	Saturates [%]	14.68
	Aromatics [%]	43.28
	Resins [%]	32.85
	Asphaltenes [%]	9.19

**Table 3:** Fundamental properties of penetrative rejuvenator penetrative rejuvenator (PR).

Items		Results
Physical properties	pH values	5.6
	Density [g·mL^−1^]	0.97
	Viscosity (25 °C) [cP]	478
Chemical compositions	Saturates [%]	25.57
	Aromatics [%]	29.91
	Resins [%]	39.98
	Asphaltenes [%]	4.41

**Table 4:** Test samples and labels.

Copolymers	Temperature [°C]	Time [h]	UV Intensity [uw/cm^2^]	Rejuvenated Labels
Origin-SBS	N/A	N/A	N/A	Origin-SBS-R
UV-1	50	24	998	UV-1-R
UV-5	50	120	998	UV-5-R
TFOT-1	163	1	N/A	TFOT-1-R
TFOT-5	163	5	N/A	TFOT-5-R

**Table 5:** Labels of asphalt samples.

Copolymers	MAAC	MARC
Origin-SBS	SBS-A	N/A
UV-1	UV-1-A	UV-1-R
UV-5	UV-5-A	UV-5-R
TFOT-1	TFOT-1-A	TFOT-1-R
TFOT-5	TFOT-5-A	TFOT-5-R

**Table 6:** Peaks and assignments.

Wavenumber (cm^−1^)	Assignments	Origin of the Chemical Structures
3460	Hydroxyl (O–H) stretching	Carboxyl acids, alcohols, hydroperoxides and etc.
2919	Methylene C–H anti-symmetry stretching	Methylene units
2845	Methyl C–H symmetry stretching	Methylene units
1780	Carbonyl (C=O) stretching	Anhydrides, lactones, peracids and so on.
1725	Carbonyl (C=O) stretching	Aliphatic ketones, aldehydes, etc.
1700	Carbonyl (C=O) stretching	α, β unsaturated acids, ketones, aldehydes and acetophenone groups
1160	Ether bond (–O–) stretching	Fatty ethers or aromatic ethers
1030	Sulfoxide (S=O) stretching	Thioether and sulfoxide units
967	C-H bending vibration	1-4 trans olefinic groups
910	C-H bending vibration	1-2 vinyl olefinic groups

**Table 7:** Structural indices of samples.

	I_CH=CH_	I_C=O_	I_S=O_
UV-5-A	0.01915	0.03857	0.02696
UV-5-R	0.02534	0.04003	0.02061
UV-1-A	0.03273	0.02088	0.02159
UV-1-R	0.03934	0.03543	0.02075
TFOT-5-A	0.02520	0.02769	0.02054
TFOT-5-R	0.02970	0.03283	0.01748
TFOT-1-A	0.03225	0.01968	0.01957
TFOT-1-R	0.03560	0.02840	0.01456
SBS-A	0.03565	0.01773	0.01987

## References

[1] Khodaii A., Mehrara A. (2009). Evaluation of permanent deformation of unmodified and SBS modified asphalt mixtures using dynamic creep test. Constr. Build. Mater..

[2] Larsen D.O., Alessandrini J.L., Bosch A., Cortizo M.S. (2009). Micro-structural and rheological characteristics of SBS-asphalt blends during their manufacturing. Constr. Build. Mater..

[3] Duan S., Muhammad Y., Li J., Maria S., Meng F., Wei Y., Su Z., Yang H. (2019). Enhancing effect of microalgae biodiesel incorporation on the performance of crumb Rubber/SBS modified asphalt. J. Clean. Prod..

[4] Zhang H., Jia X., Yu J., Xue L. (2013). Effect of expanded vermiculite on microstructures and aging properties of styrene–butadiene–styrene copolymer modified bitumen. Constr. Build. Mater..

[5] Zeng W.B., Wan L., Peng Z.Q., Cui P.Q., Wu S.P. (2014). Effects of Various Rejuvenator Sealer Materials on Rheological Properties of Aged SBS Modified Asphalt. Key Eng. Mater..

[6] Yu J., Wang L., Zeng X., Wu S., Li B. (2007). Effect of montmorillonite on properties of styrene-butadiene-styrene copolymer modified bitumen. Polym. Eng. Sci..

[7] Lu X., Isacsson U. (1997). Influence of styrene-butadiene-styrene polymer modification on bitumen viscosity. Fuel.

[8] Wang J., Qin Y., Huang S., Xu J. (2017). Laboratory Evaluation of Aging Behaviour of SBS Modified Asphalt. Adv. Mater. Sci. Eng..

[9] Adedeji A., Grünfelder T., Bates F.S., Macosko C.W., Stroup-Gardiner M., Newcomb D.E. (1996). Asphalt Modified by SBS Triblock Copolymer: Structures and Properties. Polym. Eng. Sci..

[10] Xiang K., Wang X., Huang G., Zheng J., Huang J., Li G. (2012). Thermal ageing behavior of styrene–butadiene random copolymer: A study on the ageing mechanism and relaxation properties. Polym. Degrad. Stab..

[11] Hu X., Luo Z. (1995). Wavelength sensitivity of photooxidation of styrene-butadiene-styrene copolymer. Polym. Degrad. Stab..

[12] Singh R.P., Desai S.M., Solanky S.S., Thanki P.N. (2015). Photodegradation and stabilization of styrene–butadiene–styrene rubber. J. Appl. Polym. Sci..

[13] Gao Y., Gu F., Zhao Y. (2013). Thermal oxidative aging characterization of SBS modified asphalt. J. Wuhan Univ. Technol. Mater. Sci. Ed..

[14] Cortizo M.S., Larsen D.O., Bianchetto H., Alessandrini J.L. (2004). Effect of the thermal degradation of SBS copolymers during the ageing of modified asphalts. Polym. Degrad. Stab..

[15] Lucena M.D.C.C., Soares S.D.A., Soares J.B. (2004). Characterization and thermal behavior of polymer-modified asphalt. Mater. Res. Ibero Am. J. Mater..

[16] Wang Y., Sun L., Qin Y. (2015). Aging mechanism of SBS modified asphalt based on chemical reaction kinetics. Constr. Build. Mater..

[17] Xu J., Zhang A., Zhou T., Cao X., Xie Z. (2007). A study on thermal oxidation mechanism of styrene–butadiene–styrene block copolymer (SBS). Polym. Degrad. Stab..

[18] Cong P., Luo W., Xu P., Zhao H. (2015). Investigation on recycling of SBS modified asphalt binders containing fresh asphalt and rejuvenating agents. Constr. Build. Mater..

[19] Zhang D., Zhang H., Zhu C. (2017). Effect of different rejuvenators on the properties of aged SBS modified asphalt. Pet. Sci. Technol..

[20] Xu S., Yu J., Hu C., Qin D., Xue L. (2017). Laboratory evaluation of rejuvenation effect of reactive rejuvenator on aged SBS modified bitumen. Mater. Struct..

[21] Guo M., Tan Y., Luo D., Li Y., Farooq A., Mo L., Jiao Y. (2018). Effect of Recycling Agents on Rheological and Micromechanical Properties of SBS-Modified Asphalt Binders. Adv. Mater. Sci. Eng..

[22] Xu X., Yu J., Zhang C., Cao Z., Gu Y., Xue L. (2017). Effect of reactive rejuvenators on structure and properties of UV-aged SBS modified bitumen. Constr. Build. Mater..

[23] Simões D., Almeida-Costa A., Benta A. (2017). Preventive maintenance of road pavement with microsurfacing—An economic and sustainable strategy. Int. J. Sustain. Transp..

[24] Giustozzi F., Crispino M., Flintsch G. (2012). Multi-attribute life cycle assessment of preventive maintenance treatments on road pavements for achieving environmental sustainability. Int. J. Life Cycle Assess..

[25] Hussein I.A., Al Mehthel M.H., Wahhab H.I.A.-A., Al Idi S.H., Akhtar J.S. (2015). Sulfur Extended Polymer for Use in Asphalt Binder and Road Maintenance. U.S. Patents.

[26] Hussain M., Ghaly N., Ibrahim I. (2008). Modified Hot Mix Asphalt for Road Maintenance. World Appl. Sci. J..

[27] Liu Z.B. (2014). Asphalt Pavement Preventive Maintenance Technology Overview. Appl. Mech. Mater..

[28] Lin J., Hong J., Huang C., Liu J., Wu S. (2014). Effectiveness of rejuvenator seal materials on performance of asphalt pavement. Constr. Build. Mater..

[29] Prapaitrakul N., Freeman T.J., Glover C.J. (2005). Analyze Existing Fog Seal Asphalts and Additives: Literature Review. Asph. Emuls..

[30] Zhang J.P., Luo P.F., Xu L., Ma X.J. Laboratory Study on the Permeability and Skid Resistance of Asphalt Pavement Fog Seal Layer. Proceedings of the 15th COTA International Conference of Transportation.

[31] Xiao Y., Van De Ven M., Molenaar A., Su Z., Zandvoort F. (2011). Characteristics of two-component epoxy modified bitumen. Mater. Struct..

[32] Wang F., Zhang L., Yan B., Kong D., Li Y., Wu S. (2019). Diffusion Mechanism of Rejuvenator and Its Effects on the Physical and Rheological Performance of Aged Asphalt Binder. Materials.

[33] Klint A. (2011). Amphiphilic Surface Modification of Colloidal Silica Sols.

[34] Wang P., Dong Z.J., Tan Y.Q., Liu Z.Y. (2015). Investigating the Interactions of SARA Four-Fraction in Asphalt Binders by Molecular Simulations. Energy Fuels.

[35] Lin J., Guo P., Wan L., Wu S. (2012). Laboratory investigation of rejuvenator seal materials on performances of asphalt mixtures. Constr. Build. Mater..

[36] ASTM D2872-19 (2019). Standard test method for effect of heat and air on a moving film of asphalt (rolling thin-film oven test).

[37] ASTM G154-16 (2006). Standard Practice for Operating Fluorescent Ultraviolet (UV) Lamp Apparatus for Exposure of Nonmetallic Materials.

[38] Pereira A., Micaelo R., Quaresma L., Cidade M.T. (2016). Evaluation of Different Methods for the Estimation of the Bitumen Fatigue Life with DSR Testing. 8th RILEM International Symposium on Testing and Characterization of Sustainable and Innovative Bituminous Materials.

[39] Moyo S., Mphuthi D., Cukrowska E., Henshilwood C.S., Niekerk K.V., Chimuka L. (2016). Blombos Cave: Middle Stone Age ochre differentiation through FTIR, ICP OES, ED XRF and XRD. Quat. Int..

[40] Wu S.P., Pang L., Mo L.T., Chen Y.C., Zhu G.J. (2009). Influence of aging on the evolution of structure, morphology and rheology of base and SBS modified bitumen. Constr. Build. Mater..

[41] Hendessi S., Klerk A.D. (2016). Ozonation of Oilsands Bitumen. Energy Fuels.

[42] Wang T., Cai H.M., Zhang Y.Z. (2008). Research about the Mechanism of SBS Modified Asphalt. Pet. Asphalt.

[43] Romera R., Santamaría A., Peña J.J., Muñoz M.E., Barral M., García E., Jañez V. (2006). Rheological aspects of the rejuvenation of aged bitumen. Rheol. Acta.

[44] Zhang D., Zhang H., Shi C. (2017). Investigation of aging performance of SBS modified asphalt with various aging methods. Constr. Build. Mater..

